# Magnetoelectric Nanoparticles Enable Modulation of Cortical Networks by Low‐Intensity Static Magnetic Fields In Vitro

**DOI:** 10.1002/advs.76867

**Published:** 2026-07-31

**Authors:** Nathalia Cancino‐Fuentes, Alejandro Suarez‐Perez, Elric Zhang, Hao Ye, Marta Bonato, Joana Covelo, Vitaly Pustovalov, Anton Guimera‐Brunet, Xavi Illa, Valentin Gantenbein, Cagatay M. Oral, Marta Parazzini, Salvador Pané, Maria V. Sanchez‐Vives

**Affiliations:** ^1^ Institut d'Investigacions Biomèdiques August Pi i Sunyer (IDIBAPS) Barcelona Spain; ^2^ Facultat de Medicina i Ciències de la Salut Universitat de Barcelona (UB) Barcelona Spain; ^3^ Multi‐Scale Robotics Lab Institute of Robotics and Intelligent Systems ETH Zürich Zürich Switzerland; ^4^ CNR ‐ Istituto di Elettronica e di Ingegneria dell'Informazione e delle Telecomunicazioni Milano Italy; ^5^ Institut de Microelectrònica de Barcelona (IMB‐CNM) CSIC Bellaterra Spain; ^6^ Centro de Investigación Biomédica en Red de Bioingeniería, Biomateriales y Nanomedicina Instituto de Salud Carlos III Bellaterra Spain; ^7^ ICREA Barcelona Spain

**Keywords:** cerebral cortex, in vitro, magnetoelectric nanoparticles, neuromodulation, slow oscillations, slow waves, static magnetic stimulation

## Abstract

Achieving precise and minimally invasive control of brain activity remains a major challenge in neuroscience, with current non‐invasive techniques offering limited spatial and temporal resolution. Here, this study investigated whether magnetoelectric nanoparticles (MENPs) can extend the effective neuromodulation range of static magnetic fields, enabling modulation under intensities (<100 mT) that are otherwise biologically inert. To this end, MENPs comprising a cobalt ferrite magnetostrictive core and a piezoelectric barium titanate shell were fabricated. Using magnetoelectric modeling, the non‐negligible electric fields generated by individual MENPs under magnetic‐field strengths used experimentally were estimated. The ability of MENPs to modulate cortical network activity in spontaneously rhythmic cortical slices under low‐intensity static magnetic fields was tested. Using electrophysiological recordings, network activity was directly measured before, during, and after stimulation. Magnetic fields alone did not alter neuronal activity at the intensities used. In contrast, in the presence of MENPs, the same fields activated the network, enhancing the frequency of spontaneous rhythmic activity and thus network excitability. These findings demonstrate that MENPs lower the threshold for magnetic neuromodulation, providing a mechanistic link between weak magnetic inputs and network‐level brain activity. This work position MENPs as a promising strategy for the wireless modulation of neuronal network dynamics, with the potential to reach deep brain circuits.

## Graphical abstract

Neuromodulation strategies are increasingly needed to stimulate, restore, or replace altered neuronal activity in disease. Cobalt ferrite‐barium titanate (CFO–BTO) magnetoelectric nanoparticles convert low‐intensity static magnetic fields into local electrical modulation of cortical networks. In cortical slices, magnetic fields below 100 mT are ineffective alone, but with magnetoelectric nanoparticles they reversibly enhance slow‐wave activity, establishing a wireless strategy for minimally invasive neuromodulation.

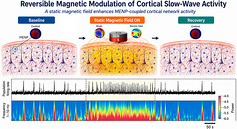

## 1 Introduction

Transcranial static magnetic field stimulation (tSMS) is a non‐invasive brain stimulation technique that employs focal static magnetic fields (SMFs) generated by a cylindrical neodymium permanent magnet placed over the scalp [1]. Unlike the dynamic magnetic fields produced by the well‐established non‐invasive brain stimulation modality, transcranial magnetic stimulation (TMS), the magnetic field in tSMS remains constant in both intensity and direction over time [2]. Several studies have demonstrated the potential of tSMS to modulate cortical excitability [1, 2, 3, 4, 5, 6, 7, 8, 9] and induce transient behavioral effects [5, 10, 11, 12]. These characteristics have motivated its application in clinical research for stroke rehabilitation [13], Parkinson's disease [14] and in animal models of epilepsy [15, 16, 17, 18].

When compared to other non‐invasive brain stimulation techniques that also modulate cortical excitability, such as repetitive TMS [19, 20, 21] and transcranial direct current stimulation (tDCS) [22, 23, 24, 25, 26, 27, 28], tSMS offers several advantages, including lower cost, portability, minimal operational skill, and the lack of seizure or tingling‐related side effects [13, 15]. However, like most non‐invasive brain stimulation techniques, tSMS is constrained by limited spatial resolution and focality [29], making the modulation of deep targets a challenge [30, 31]. When applied to the human scalp, the static magnetic field (SMF) intensity reaching the cortex (∼2–3 cm from the magnet surface) ranges between 120 and 200 mT, decreasing rapidly with distance [32]. While the strength of the applied field can be enhanced by increasing the magnet diameter or thickness, there is a tradeoff between overall strength and focality [32, 33]. As such, there is a need to develop alternative stimulation techniques able to act with spatial precision on target locations, including subcortical structures. Magnetoelectric nanoparticles (MENPs) constitute one such strategy.

MENPs are nanostructures that comprise a magnetostrictive core (e.g., CoFe_2_O_4_) coated with a piezoelectric shell (e.g., BaTiO_3_) [34]. When exposed to relatively low magnetic fields (<∼ 1000 Oe), the magnetostrictive core undergoes a non‐zero strain that propagates to the shell, generating a local electric field of the order of 1000 V/m. The magnetic field intensity required for brain stimulation with MENPs is substantially lower than that used in conventional TMS [35]. Since MENPs act as transducers, otherwise sub‐threshold or non‐interacting fields are converted into physiologically relevant local electric fields. Previous studies have shown that 30 nm MENPs are able to cross the blood‐brain barrier by application of a remote magnetic field gradient [36, 37], without inducing neuronal death in the affected brain areas [36]. Additionally, MENPs exhibit clearance rates of 90% and 86% for diameters of 10 and 30 nm, respectively [38], and have been shown to be biocompatible [39], presenting no significant toxicity at a concentration of 50 mg ml^−1^ [40]., which supports their potential use in biomedical applications [39]. As such, MENPs could be administered intravenously or intranasally and be directed to a target region through a magnetic field gradient [35], thereby allowing the wireless transmission of electrical signals to deep brain regions in response to an external magnetic field [41].

To date, the neuromodulatory potential of MENPs has only been tested in vitro and in vivo in animal models [35, 41, 42, 43]. Nguyen et al. [35] demonstrated that MENPs activation with weak external magnetic fields can evoke cortical activity in individual neurons in cortical slices (750–875 Oe single unipolar 200‐ms square pulse) and induce network activation in a large‐scale network in vivo (350–450 Oe at 5, 10 or 20 Hz) in a stimulus‐response paradigm. Kozielski et al. [41] reported that MENPs activated by a small alternating current (AC) magnetic field (∼60 Oe at 140 Hz) with a relatively large SMF bias (∼2000 Oe) were able to locally modulate neuronal activity in vitro and in vivo, enough to alter animal behavior. In hippocampal cell cultures, MENPs induced action potentials when activated with an AC magnetic field (1200 Oe at 20 Hz [42] and 1700 Oe at 50 Hz [43]) and inhibited action potentials following an SMF [43]. However, all these studies relied on the detection of calcium indicators or behavioral readouts, which provide indirect measures of neuronal activity, and the online effects of MENP‐mediated neuromodulation on emergent physiological activity under an SMF remain unexplored.

Here, we aimed to explore the mechanisms underlying network‐level modulation of cortical rhythmic activity through MENPs stimulation when activated with low‐intensity SMF. To do so, we used cortical slices expressing spontaneous slow‐wave activity. Slow‐wave activity is generated by the cortex during slow‐wave sleep and deep anesthesia [44, 45, 46], but it can also emerge during wakefulness in perilesional tissue and other pathological conditions (e.g., Massimini et al. [47]), making its targeted local modulation highly clinically relevant. We applied a neodymium static magnet before and after application of MENPs with a CoFe_2_O_4_ (CFO) core and a BaTiO_3_ (BTO) shell. The cortical network electrophysiological activity was measured online using microelectrode arrays, which provide a direct measure of brain activity. Our findings reveal that under small (<100 mT) static magnetic fields, MENPs significantly increase the excitability of the network and the frequency of spontaneous slow oscillations. This modulation was reversible and independent of field polarity, consistent with a magnetoelectric mechanism rather than passive magnetic field effects. No significant modulation was observed with magnetic fields alone (<100 mT) in the absence of MENPs.

## 2 Results

### 2.1 Magnetoelectric Characterization and Computational Modeling of MENPs

#### 2.1.1 Magnetoelectric Nanoparticle Characterization

**FIGURE 1:**
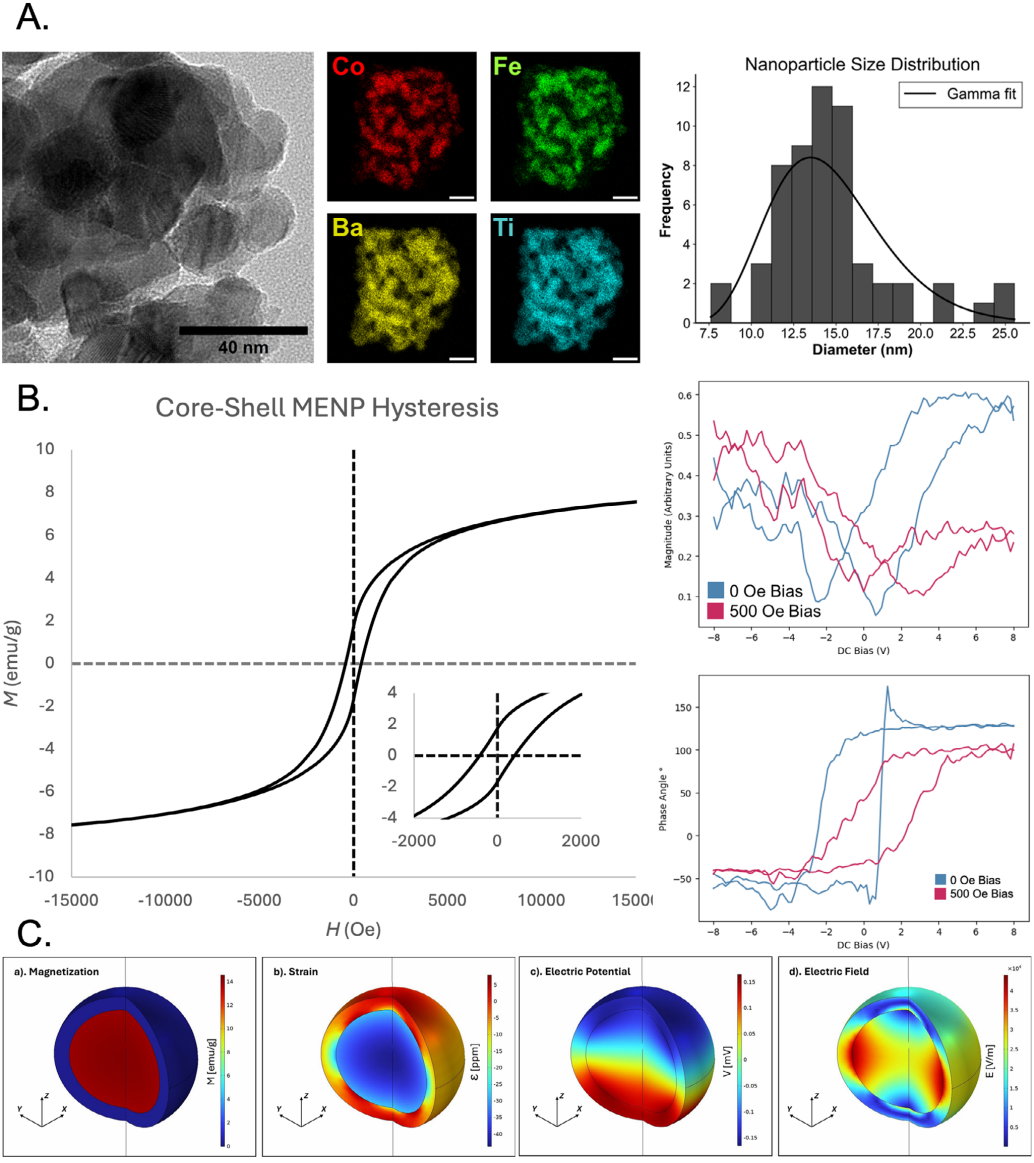
Characterization of the core‐shell magnetoelectric nanoparticles. (A) High‐Angle Annular Dark‐Field (HAADF) images with EDX overlay showing composition of core and shell elements (scale bar 40 nm). Histogram of the nanoparticle size distribution, as measured by transmission electron microscopy (TEM). (B) Magnetic hysteresis curve of the core‐shell nanoparticles with an inset of the low field initial susceptibility. Also shown are magnitude and phase‐angle piezo force microscopy measurements of the core‐shell nanoparticles showing typical shift in piezoresponse due to applied magnetic bias. (C) Simulation plots of the magnetoelectric effect elicited by 93.4 mT fields showing distribution of (a) magnetization M (emu/g), (b) strain ε (ppm), (c) electric potential V (mV) and (d) electric field E module (V/m) in 2D axisymmetric CFO‐BTO core–shell nanoparticles (Ø core diameter = 15 nm and thickness shell = 2 nm).

The MENPs used in this study consisted of a cobalt ferrite magnetostrictive core combined with a thin piezoelectric barium titanate shell. Prior to use in tissue, the nanoparticles were characterized using a variety of techniques to verify their chemical composition, morphology, colloidal behavior, and magnetoelectric response. Chemical composition and nanoscale morphology were assessed by transmission electron microscopy (TEM) and energy‐dispersive X–ray spectroscopy (EDX) elemental mapping (Figure 1A). While the TEM images alone do not fully resolve a sharp core–shell interface, the EDX elemental maps demonstrate co‐localization of CFO‐ and BTO‐associated elements, consistent with the intended core–shell particle architecture.

To further characterize the exact nanoparticle suspension used in the slice experiments, dynamic light scattering (DLS) and zeta potential measurements were performed in the same phosphate‐buffered saline (PBS) solution used for tissue delivery (Figure S1). The DLS measurements revealed marked differences between uncoated and PEG (polyethylene glycol)‐coated MENPs. Uncoated MENPs exhibited broad multimodal size distributions with dominant micron‐scale aggregates (∼1.3 µm intensity peak), indicating substantial aggregation under physiological ionic conditions. In contrast, PEG‐coated MENPs displayed a narrow number‐weighted hydrodynamic size distribution centered at ∼50–70 nm, consistent with the primary nanoparticle population, together with an intensity‐weighted distribution centered at ∼300–400 nm, indicative of small, stable sub‐micron clusters in suspension. Zeta potential measurements showed a shift from approximately −7 mV for uncoated MENPs to ∼−26 mV following PEG functionalization, consistent with improved colloidal stability and reduced aggregation propensity in PBS. Together, these data establish that the experimental nanoparticle system is best described as a stable colloidal ensemble composed of primary nanoparticles and small aggregates, rather than isolated monodisperse particles.

The magnetoelectric response was confirmed via piezoresponse force microscopy (PFM), following the procedure outlined in our previous studies [48, 49]. A pronounced shift in the piezoelectric phase response was observed upon application of a magnetic field bias (Figure 1B), confirming strain‐mediated magnetoelectric coupling between the CFO and BTO phases. From these measurements, the magnetoelectric coefficient was estimated as *a_ME_* =  196.67 × 10^4^ mV cm^−1^Oe^−1^.

#### 2.1.2 Computational Model

To evaluate the local electromechanical transduction generated by MENPs under magnetic stimulation, a finite‐element multiphysics model was implemented using an idealized single‐particle core–shell geometry representative of the experimentally measured nanoparticle size scale. The simulation was not intended to quantitatively reconstruct the full experimental suspension, which contains polydispersity and small nanoparticle clusters, but rather to provide a first‐order, order‐of‐magnitude estimate of the achievable local electric fields generated by magnetoelectric coupling at the particle scale.

Under the experimentally measured static magnetic field of 93.4 mT, the simulation predicted the key magnetoelectric parameters within a representative nanoparticle. The magnetization profile (Figure 1C(a)) showed that the CFO core reaches approximately 14 emu/g. This magnetization induced magnetostriction in the core, with compression along the applied field direction (z‐axis, up to −40 ppm) and expansion in the other two perpendicular directions (up to +5 ppm), as shown in Figure 1C(b). The mechanically induced strain transferred to the BTO shell generated a clear dipolar electric potential distribution (Figure 1C(c)), with potential values ranging from −0.2 mV at one pole to +0.2 mV at the opposite pole. Correspondingly, the electric field magnitude (Figure 1C(d)) reached maximum values of approximately 4.5×10^4^ V/m at the core‐shell interface, decaying rapidly with distance from the nanoparticle surface.

These simulations provide a nanoscale description of the magnetoelectric transduction process within MENPs under experimentally relevant magnetic stimulation conditions. The model predicts that magnetically induced strain in the CFO core generates localized electric potentials and strong interfacial electric fields in the surrounding BTO shell, supporting the proposed mechanism of nanoparticle‐mediated electrical modulation. While the simulations employ an idealized single‐particle core–shell geometry, the experimentally measured colloidal characterization demonstrates that the nanoparticle suspension is predominantly composed of primary nanoparticles together with stable sub‐micron clusters, providing an experimentally grounded size scale for the modeled system. Collectively, these results establish the physical plausibility and characteristic spatial scale of the electric fields generated by MENPs under magnetic stimulation.

### 2.2 Low‐Intensity DC Magnetic Stimulation (<100 mT) Does Not Modulate Slow‐Oscillatory Activity in the Absence of MENPs

**FIGURE 2:**
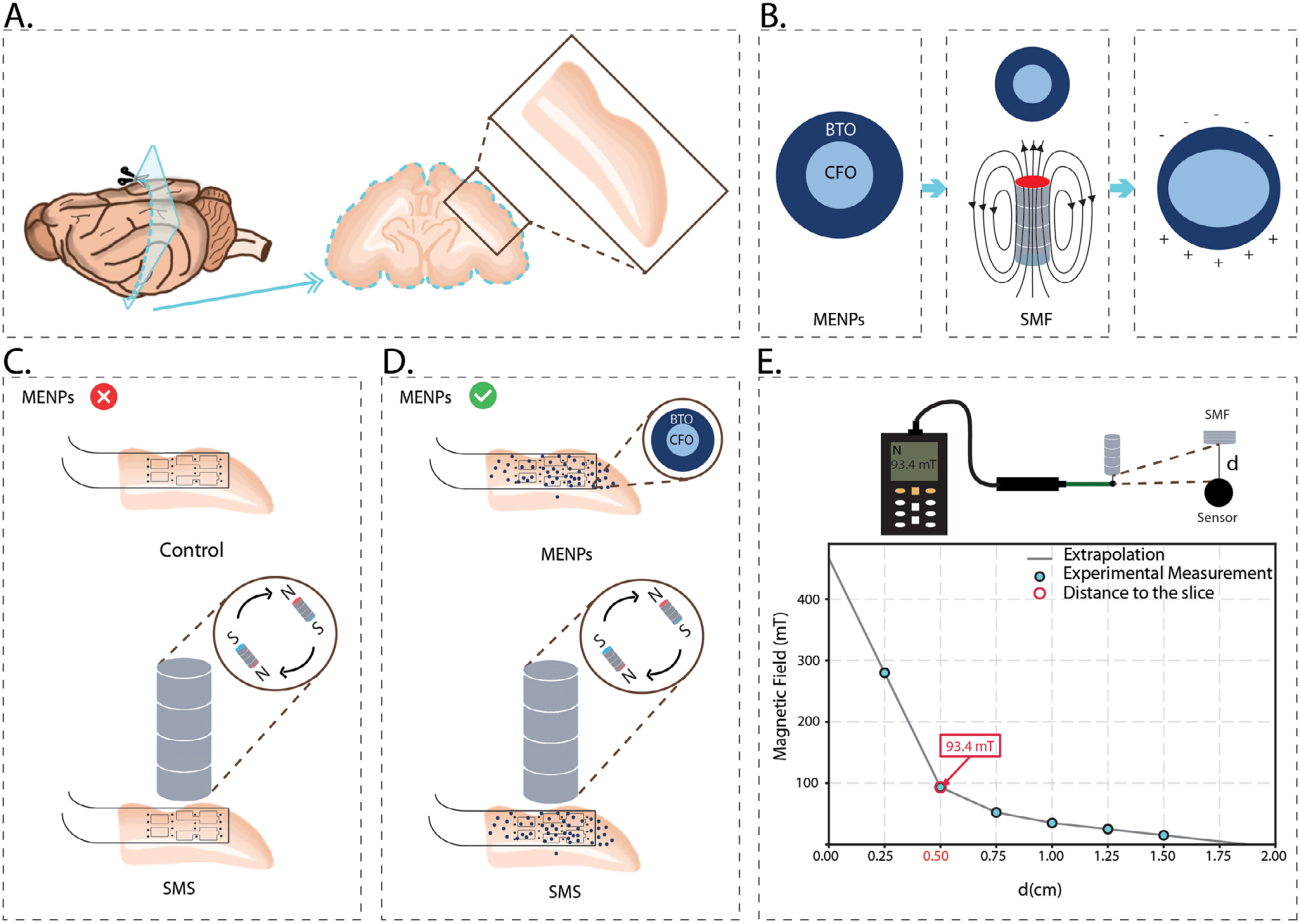
Methodological overview of the experimental approach. (A) Schematic representation of the coronal slicing procedure used to obtain 400‐µm‐thick cortical slices. (B) Illustration of the magnetoelectric coupling mechanism: the CoFe_2_O_4_ (CFO) magnetostrictive core deforms under static magnetic fields (SMF), transferring strain to the BaTiO_3_ (BTO) piezoelectric shell and generating a local electric dipole. (C) Control protocol without MENPs, showing baseline recording, static magnetic stimulation (SMS), and polarity switching (north/south). (D) Experimental protocol with MENPs applied to the slice, followed by SMS with alternating north and south polarities. (E) Characterization of the neodymium magnet used for stimulation, including the measurement setup, distance to the slice (∼0.5 cm), and corresponding magnetic field intensity (93.4 mT).

Previous studies have reported that static magnetic fields of moderate intensity (∼300–500 mT) can influence cortical excitability in both rodents and primates, reducing neuronal firing, delaying epileptiform activity, and altering sensory responses [50]. In contrast, the effects of low‐intensity DC magnetic fields (<100 mT) on spontaneous cortical activity remain unclear. To determine whether such low‐intensity static magnetic stimulation (SMS) can modulate ongoing cortical activity on its own, we applied low‐intensity SMS to 400‐µm coronal cortical slices, obtained as illustrated in Figure 2A. The subsequent panels in Figure 2B–E summarize the experimental configuration and stimulation protocol used in this study, with full procedural details available in the Methods section. We applied low‐intensity SMS to slices of ferret primary visual cortex in the absence of MENPs.

**FIGURE 3:**
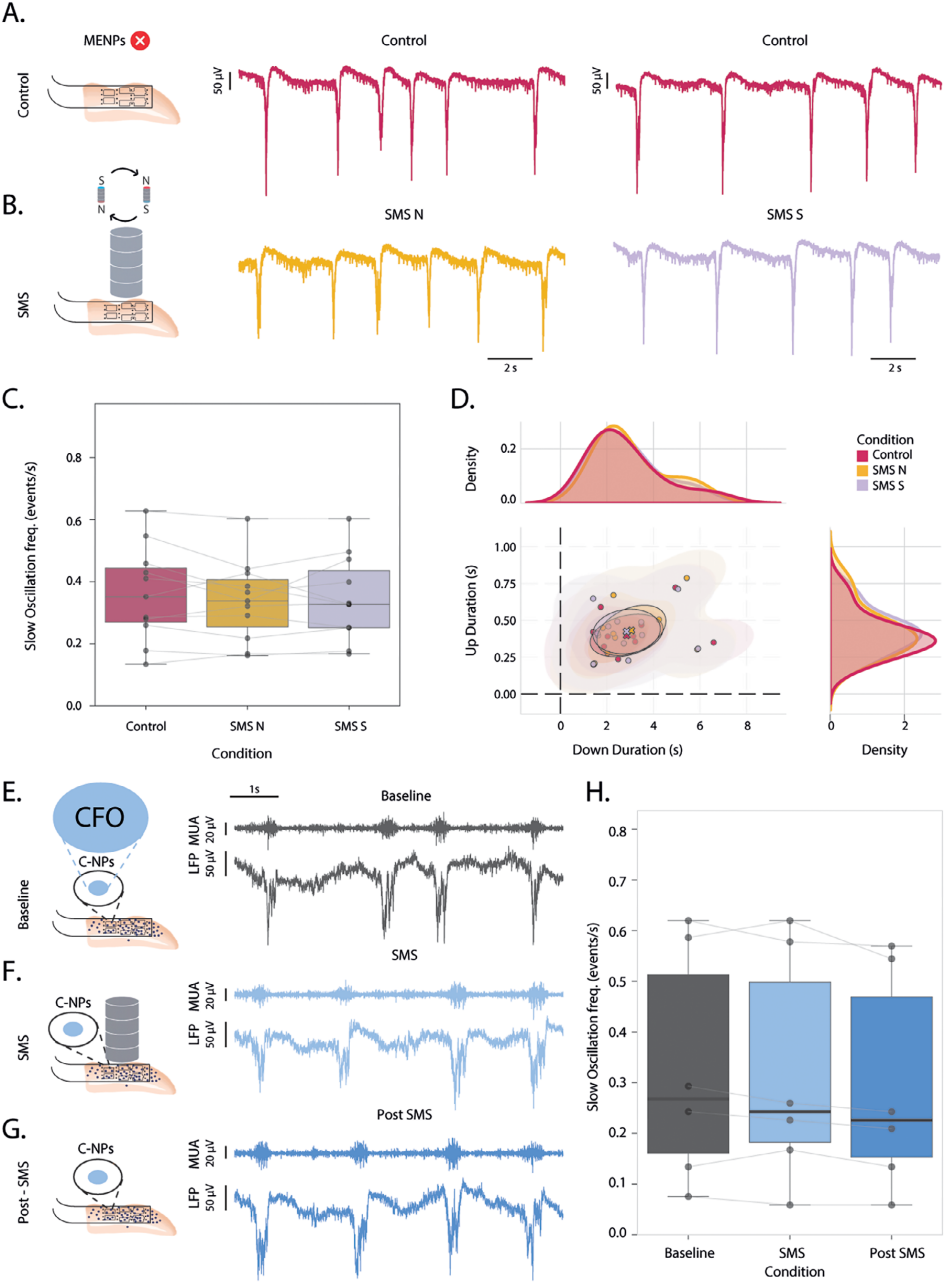
Low‐intensity DC magnetic stimulation (<100 mT) does not modulate slow‐oscillatory activity in cortical slices in the absence of magnetoelectric coupling. (A) Representative examples of baseline spontaneous slow oscillations in slices without MENPs and before low‐intensity SMS application. Left: schematic of the recording configuration. Center and right: example local field potential (LFP) traces of spontaneous activity aligned to the locations where north and south polarity low‐intensity SMS would later be applied. (B) Local field potential activity during low‐intensity SMS application. Left: schematic of the recording condition. Center and right: representative LFP traces recorded during north‐ and south‐polarity low‐intensity SMS, showing preservation of slow‐oscillatory activity. (C) Boxplots of slow‐oscillation frequency (Up events/s) for baseline, north‐polarity stimulation, and south‐polarity stimulation conditions. No significant differences were observed across conditions (N_slices_ = 11 paired; Friedman repeated‐measures test followed by paired Wilcoxon signed‐rank post hoc tests with BH‐FDR correction; all adjusted *p* > 5.00 × 10^−2^). (D) Two‐dimensional representation of Up‐ versus Down state durations for each condition, with marginal density plots for Up‐state durations (right) and Down‐state durations (top). Up‐ and Down‐state durations and densities remain unchanged across conditions (N_slices_ = 11; paired Wilcoxon signed‐rank tests on slice‐averaged values; all *p* > 5.00 × 10^−2^). (E–G) Control experiments using cobalt ferrite (CFO) cores lacking the piezoelectric BTO shell. Left: schematic of the recording configuration. Right: representative multi‐unit activity (MUA, top) and local field potential (LFP, bottom) recordings obtained during baseline (E), static magnetic stimulation (F), and post‐stimulation recovery (G), showing preserved spontaneous slow‐oscillatory dynamics across conditions. (H) Boxplots of slow‐oscillation frequency (Up events/s) during baseline, stimulation, and post‐stimulation conditions in slices incubated with CFO cores alone. No significant differences were detected between conditions (N_slices_ = 6; linear mixed‐effects models with BH‐FDR correction; all adjusted *p* > 5.00 × 10^−2^).

We first confirmed that cortical slices exhibited stable spontaneous slow oscillations under control conditions [51, 52], network activity that has been demonstrated to be highly similar to that observed in slow wave sleep or anesthesia [53, 54]. Exposure to the neodymium magnet, whether in north or south polarity, did not alter the characteristic spatiotemporal patterns of slow‐oscillatory activity (Figure 3A,B). Quantification of slow oscillation frequency revealed no significant effect of magnetic‐field polarity in control slices lacking MENPs (Figure 3C). Slow‐oscillation frequencies remained comparable across baseline, north‐polarity stimulation, and south‐polarity stimulation conditions (control: 0.36 ± 0.15 events/s; control north: 0.34 ± 0.13 events/s; control south: 0.35 ± 0.14 events/s; N_slices_ = 11 paired). A Friedman repeated‐measures test showed no overall differences between conditions (χ^2^ = 2.48, p = 2.9 × 10^−1^), and post hoc paired Wilcoxon signed‐rank tests with Benjamini–Hochberg false discovery rate (BH‐FDR) correction confirmed the absence of significant pairwise effects (all adjusted *p* > 5.00 × 10^−2^).

To directly address potential effects on neuronal spiking activity, we quantified multi‐unit activity (MUA) firing rates during both Up and Down states under the same stimulation conditions. Firing rate was computed as the average logMUA within each state (see Methods). During Up states, relative firing rate values were 1.43 ± 0.46 a.u. (control), 1.46 ± 0.53 (control north), and 1.40 ± 0.50 (control south) (N_slices_ = 11; Mann–Whitney U tests, all pairwise comparisons p > 5.00 × 10^−2^). During Down states, relative firing rate values were 0.189 ± 0.060 (control), 0.191 ± 0.059 (control north), and 0.19 ± 0.08 (control south) (N_slices_ = 11; Mann–Whitney U tests, all pairwise comparisons p > 5.00 × 10^−2^). No significant differences were detected across conditions in either state.

Similarly, analysis of Up and Down state durations showed no significant changes during low‐intensity SMS relative to baseline conditions (Figure 3D). Down state durations were 2.85 ± 1.57 s (control), 3.05 ± 1.48 s (control north), and 2.85 ± 1.45 s (control south), while Up state durations were 0.40 ± 0.15 s, 0.43 ± 0.17 s, and 0.42 ± 0.15 s, respectively (N_slices_ = 11; paired Wilcoxon signed‐rank tests on slice‐averaged values, all *p* > 5.00 × 10^−2^).

Together, these results indicate that static DC magnetic fields below 100 mT do not modulate the spontaneous slow‐oscillation dynamics of cortical slices in the absence of MENPs, and that neither magnetic‐field polarity exerts detectable effects on the network‐level firing activity or spike rates under these conditions.

### 2.3 Effects of CFO Cores on Slow Oscillatory Activity Without and With Static Magnetic Stimulation

In order to investigate whether CFO cores had any effect on the cortical rhythmic activity, we performed control experiments using CFO magnetostrictive cores suspended in PBS without the piezoelectric BTO shell (Figure 3E–H). Cortical slices exhibited stable spontaneous slow oscillations during baseline. CFO cores were then applied onto the cortical slices (N_slices_ = 7, Figure 3E–G), without producing significant changes in spontaneous slow oscillatory activity (slow oscillation frequency 0.35 ± 0.21 Hz under control conditions versus 0.34 ± 0.23 Hz after CFO core application; p = 0.68, Wilcoxon signed‐rank test).

Static magnetic stimulation was then applied to slices treated with CFO cores (*n* = 6 slices; Figure 3E–G). Quantification of slow‐oscillation frequency revealed no significant effect of stimulation, with frequencies of 0.33 ± 0.23 Hz before stimulation and 0.32 ± 0.23 Hz during stimulation (Figure 3H). Measurements were repeated within each slice during magnet ON/OFF cycles, and repeated observations were accounted for using a linear mixed model (LMM coefficient = −0.014, pFDR = 2.84 × 10−1; *n* = 6 slices). Likewise, no significant difference was observed between stimulation and post‐stimulation conditions (0.32 ± 0.23 Hz versus 0.29 ± 0.21 Hz; LMM coefficient = −0.021, pFDR = 2.84 × 10−1; n = 6 slices).

These results indicate that CFO cores alone are insufficient to modulate slow cortical oscillations under static magnetic stimulation. This supports the interpretation that the effects observed below with CFO‐BTO MENPs depend on the presence of the piezoelectric BTO shell and are consistent with magnetoelectric coupling.

### 2.4 Effects of MENPs on Baseline Slow Oscillations in the Absence of Magnetic Stimulation

Having established that low‐intensity SMS alone does not modify spontaneous SO, we next examined whether MENPs deposition affected baseline cortical dynamics in the absence of magnetic stimulation. Although MENPs have previously been reported as biocompatible in cultured neurons, brain slices, and in vivo preparations [36, 39, 40, 55], it remained essential to determine how their deposition onto an active cortical network influences the ongoing oscillatory dynamics prior to magnetoelectric activation.

**FIGURE 4:**
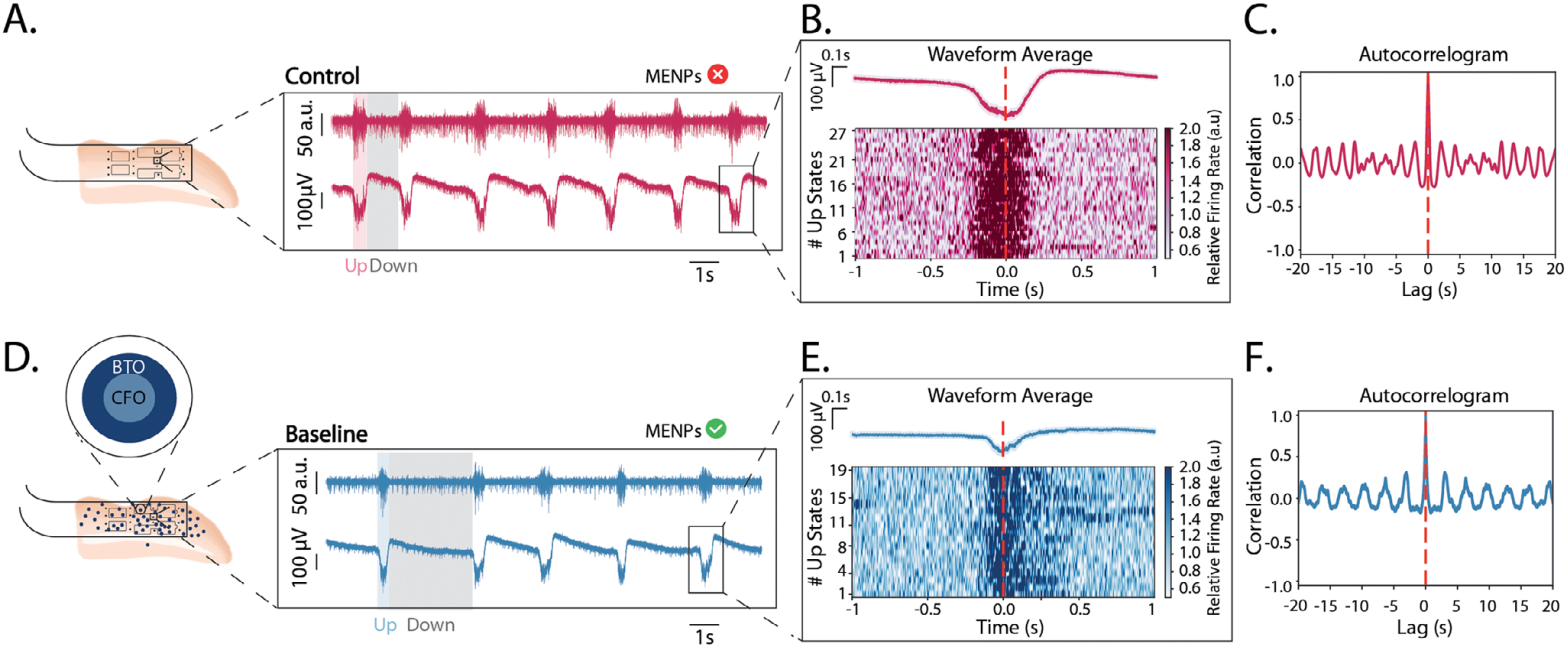
Inactive MENPs preserve the overall slow‐oscillatory pattern. (A) Illustration of the slice and recording configuration, with representative LFP trace (bottom) and corresponding multi‐unit activity (MUA, top) showing alternating Up (pink) and Down (grey) states under baseline conditions. (B) Raster plot of instantaneous firing rates for individual Up states aligned to time 0, with the average Up state waveform shown above. (C) LFP autocorrelogram illustrating the periodicity and stability of spontaneous slow oscillations at baseline. (D–F) Same analyses performed one hour after application of 12 µg of inactive MENPs (blue). (D) Slice cartoon and representative LFP/MUA traces showing preserved Up/Down state structure. (E) Raster plot and average Up state waveform demonstrating conserved firing‐rate dynamics. (F) Autocorrelogram confirming that the oscillatory rhythm remains unchanged following inactive MENP application.

To isolate the intrinsic effects of MENPs from magnetic stimulation, 12 µg of inactive MENPs (core‐shell) suspended in PBS were applied onto cortical slices exhibiting stable slow oscillations (N_slices_ = 6), and activity was recorded after a one‐hour recovery period. The control oscillatory frequency (0.36 ± 0.18 s) became 0.21 ± 0.09 Hz (*p* = 0.06; Bonferroni‐corrected Wilcoxon signed‐rank test; N_slices_ = 6) (Figure 4). Even when there was a trend toward a decrease in slow oscillation frequency, it was not statistically significant. This is in agreement with what has been described for the application of PBS with CFO‐cores (no shells) above. These results confirm that inactive MENPs are well tolerated by cortical tissue, the overall slow‐oscillation pattern was preserved in the presence of nanoparticles, and this finding establishes a stable post‐MENP baseline suitable for subsequent stimulation experiments.

### 2.5 MENPs Activated by DC Magnetic Fields Modulate Slow‐Oscillatory Activity

Having established that inactive MENPs do not perturb baseline slow‐oscillatory dynamics, we then asked whether MENPs could serve as localized mediators of neuronal stimulation under low‐intensity SMS. The central question was whether cortical activity could be modulated following MENPs application. Previous studies have shown that MENPs can influence neural activity in vitro and in vivo, for example by inducing calcium transients in cultured networks [42, 43] or evoking cortical responses in mice [35, 37]. Here, we directly recorded electrophysiological activity from MENP‐activated cortical slices under low‐intensity SMS, allowing us to assess network‐level modulation.

**FIGURE 5:**
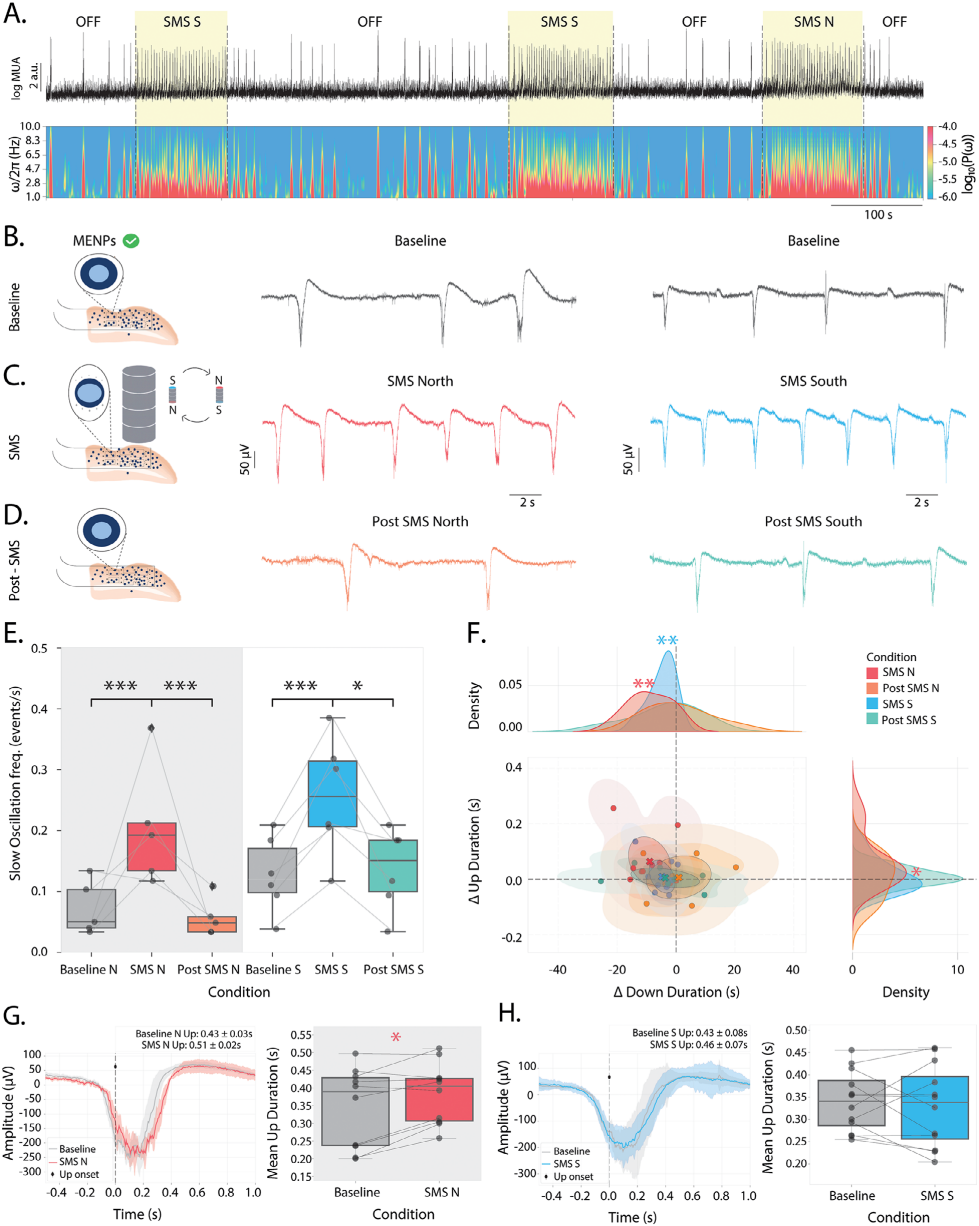
MENPs activated by static magnetic fields modulate slow‐oscillatory activity. (A) Example multi‐unit activity shown as logMUA (top) and corresponding 1–10 Hz spectrogram (bottom) during periods without stimulation (OFF) and during MENP activation with a DC magnetic field (ON, yellow shading). (B–D) Representative local field potential (LFP) recordings during baseline (B), MENP stimulation (C), and post‐stimulation (D). Each panel includes a schematic of the experimental configuration (left) and LFP traces for north and south polarities (right). Baseline activity is shown in black; MENP stimulation in red (north) and blue (south); post‐stimulation in orange (north) and green (south). (E) Boxplots of slow‐oscillation frequency (Up events/s) during baseline, stimulation, and post‐stimulation conditions for north‐ and south‐polarity SMS in slices containing MENPs. Both polarities significantly increased slow‐oscillation frequency during stimulation, and the effect reversed following magnet removal (N_slices_ = 5–6; linear mixed‐effects models with BH‐FDR correction). (F) Two‐dimensional representation of relative changes in Up state duration (*x*‐axis) and Down state duration (*y*‐axis) compared with baseline, with density projections along each axis. Colors follow the figure legend. (G) Representative averaged Up state waveforms aligned to Up state onset for baseline (grey) and MENP‐north activation (red), with paired comparisons of mean Up state duration (right). (H) Same analysis for south‐polarity stimulation (blue), showing no significant change in Up‐state duration relative to baseline (N_trials_ = 12, paired Wilcoxon signed‐rank test, p = 4.70 × 10^−1^; N_slices_ = 6). Data are presented as mean ± SD. ^*^ *p* < 5.00 × 10^−2^; ^**^ *p* < 1.00 × 10^−2^; ^***^ *p* < 1.00 x 10^−3^.

Upon application of low‐intensity DC magnetic fields in the presence of MENPs, we observed robust modulation of spontaneous slow oscillations. Local field potentials (LFPs) revealed a pronounced increase in network activity during stimulation, marked by heightened firing in the multi‐unit activity and enhanced power in the low‐ and high‐frequency bands (Figure 5A). These effects were immediate upon magnet positioning, indicating direct and rapid activation of the cortical network by MENPs.

Population‐level analysis confirmed these observations. Both north‐ and south‐polarity stimulation increased the slow‐oscillation frequency (Up events/s; Figure 5E). Under north‐polarity stimulation, frequencies increased from 0.07 ± 0.04 to 0.21 ± 0.10 events/s during stimulation (LMM coefficient = 0.14, pFDR = 3.35 × 10^−6^; N_slices_ = 5). Under south‐polarity stimulation, frequencies increased from 0.13 ± 0.06 to 0.26 ± 0.10 events/s during stimulation (LMM coefficient = 0.14, pFDR = 1.06 × 10^−10^; N_slices_ = 6). The effect reversed following magnet removal for both polarities (north: coefficient = −0.14, pFDR = 1.72 × 10^−6^; south: coefficient = −0.119, pFDR = 3.10 × 10^−2^), confirming that MENP‐mediated modulation was reversible and dynamically controlled.

To evaluate changes in the temporal structure of slow oscillations, we analyzed Up and Down state durations (Figure 5F). MENPs activation consistently shortened the Down state period for both polarities, with north stimulation reducing Down‐state duration by 8.93 ± 6.88 s (paired Wilcoxon signed‐rank test, *p* = 9.80 × 10^−3^; N_slices_ = 5) and south‐polarity stimulation reducing Down‐state duration by 5.30 ± 4.35 s (paired Wilcoxon signed‐rank test, p = 2.00 × 10^−3^; N_slices_ = 6).

The effect on Up‐state duration was polarity dependent. Stimulation with north polarity led to a modest but significant increase in Up state duration compared with baseline (Baseline N: 0.35 ± 0.11 s; low‐intensity SMS N: 0.38 ± 0.09 s; paired Wilcoxon signed‐rank test, *p* = 4.88 × 10^−2^; N_slices_ = 5; Figure 5G), consistent with a slightly more sustained depolarized state. In contrast, south‐polarity stimulation did not produce a significant change in Up state duration (Baseline S: 0.34 ± 0.07 s; low‐intensity SMS S: 0.33 ± 0.09 s; *p*  =  4.70 × 10^−1^, N_slices_  =  6; Figure 5H). These polarity‐specific effects were visualized in the 2D Up–Down duration plane (Figure 5F) and were fully reversible after magnet removal, indicating that MENP‐mediated modulation of Up state duration is dynamic and dependent on magnetic‐field polarity.

Taken together, these results demonstrate that MENPs enable low‐intensity SMS to selectively reshape cortical slow oscillations. Activation of MENPs compresses Down states for both polarities, while selectively prolonging Up states under north‐polarity stimulation. This highlights MENPs as a versatile and reversible tool for localized neuromodulation without genetic or chemical intervention.

## 3 Discussion

Non‐invasive brain stimulation (e.g., TMS, tDCS) can modulate brain activity, either by altering neuronal firing or influencing the networks’ intrinsic excitability and plasticity [30], while avoiding potential complications of invasive procedures [56, 57]. However, these techniques lack spatial resolution and focality [29], with precision and efficacy declining markedly with increasing depth [58]. For instance, a typical TMS field penetrates only about 1–1.5 cm and achieves a spatial resolution of 3–5 cm [35, 59, 60]. Considering the crucial role of subcortical brain areas for motor and cognitive processes, neurodegenerative diseases, post‐lesion recovery and adaptive reorganization, it is essential to develop non‐invasive approaches capable of reaching deeper brain structures [61]. Some of the novel efforts toward this goal include transcranial interference stimulation [61, 62, 63, 64, 65, 66, 67] and focused ultrasound stimulation [29, 68, 69, 70, 71, 72, 73]. Here, we show that weak static magnetic fields (<100 mT) become physiologically effective neuromodulatory stimuli when combined with MENPs, paving the way for a new minimally invasive stimulation strategy combining tSMS with MENPs.

Our study demonstrates that in cortical slices, magnetic activation of CFO–BTO MENPs consistently increased slow‐oscillation frequency and shortened Down states, while producing smaller, polarity‐dependent changes in Up‐state structure. The same magnetic field alone had no effect on cortical dynamics. These results establish a functional principle: MENPs convert magnetic fields that are subthreshold for neuromodulation into effective electrical perturbations capable of reshaping cortical network activity. This conversion requires the interaction between CFO‐cores and BTO‐shells, and it has been proven that the presence of CFO‐cores plus magnetic fields induce no detectable changes in cortical network activity.

Static magnetic fields in the 300–500 mT range are known to suppress cortical excitability across species and preparations [1, 10, 50, 74], and even the lowest effective intensities reported in vivo typically exceed 150–200 mT. In our slices, the surface field was <93.4 mT, well below these thresholds, and, as expected, did not alter the slow oscillation when applied alone. However, once CFO‐BTO MENPs were present, the same field became strongly excitatory, producing an immediate increase in the frequency of the oscillation through a marked shortening of Down states. This divergence underscores that these MENPs fundamentally change how weak magnetic inputs interact with cortical circuits. Moreover, these changes were observed during a stimulation period of only 100 s, when tSMS requires at least 10 min of stimulation to induce significant effects [1]. As such, cortical modulation with static magnetic stimulation (SMS) combined with CFO‐BTO MENPs requires a lower SMF intensity and duration when compared to SMS alone. Consequently, we speculate that the fields generated by conventional non‐invasive magnetic stimulation techniques (tSMS, TMS) reaching deep brain structures with lower intensities might be able to modulate those regions when combined with MENPs. To increase focality, limiting the effect to deep structures, further studies should assess the feasibility of activating MENPs with other novel non‐invasive technologies, such as focused ultrasound stimulation [29, 68, 69, 70, 71, 72, 73].

Despite these advantages, our results reveal MENPs do not merely intensify the effects of SMS; they also have distinct neuromodulatory properties. First, while in tSMS the stimulation is polarity‐independent [1], MENP stimulation resulted in a polarity‐dependent effect on Up state duration, with north polarity prolonging the Up state, whereas south polarity did not. This asymmetry aligns with the vectorial nature of magnetoelectric coupling [34, 43, 75], where polarity reverses the orientation and sign of the induced electric field. Up states represent metastable attractors stabilized by recurrent excitation; even subtle depolarizing biases can extend their duration [76].

Another key difference between tSMS and MENPs activated with SMF is their effect on excitability. While tSMS has been repeatedly shown to decrease excitability [1, 2, 3, 4, 5, 6, 7, 8, 9], the increase in SO frequency observed here indicates an increase in excitability levels. These contrasting results might derive from the fact that tSMS studies usually test excitability levels following stimulation, rather than during stimulation. As illustrated in Figure 5, immediately after MENP stimulation, we sometimes observed a rebound period where excitability was decreased for several seconds before returning to baseline levels. In tSMS, the stimulation after‐effects last for several minutes [1, 7], probably due to the larger intensities and much longer (10 min vs. 100 s) stimulation period.

The excitability increase observed during CFO‐BTO MENP stimulation was similar to that previously observed in cortical slices during positive DC electric field stimulation [27, 28, 77], which represents a model for anodal tDCS. In contrast to TMS, weak electric field stimulation is subthreshold, with minor changes in membrane potential (approximately 0.5 mV per 2 V/m [77]) and spike timing being amplified by the recurrent connectivity of the cortical network [78]. When applied to SO, positive DC fields also increased SO frequency, mainly by a shortening of Down states, with Up‐state duration hardly varying, only shortening for higher field intensities (>5–6 V/m) [28].

Under a constant DC magnetic field, MENPs are expected to generate a constant DC electric field [43]. However, unlike classical DC electrical stimulation, the field generated by MENPs will not be distributed evenly across the neuron, since it will depend on the MENP placement. As such, unlike classical DC electric stimulation, which primarily depolarizes pyramidal neurons aligned with the field [77], MENPs act on all nearby cells, including both excitatory and inhibitory neurons. The fact that MENP stimulation does not require an alignment of the electric fields with the cortical columns for the field to have an effect [27] allows directionality‐independent stimulation, which is an important advantage considering cortical folding. Nevertheless, future studies should explore whether the direction of the magnetic field reaching the MENPs affects the observed modulation.

Our findings also complement and extend previous MENP studies. Earlier work demonstrated that MENPs can modulate neural activity in cell cultures or in vivo using AC (dynamic) or combined AC+DC (dynamic + static) magnetic fields, leading to depolarization, calcium spikes, or c‐Fos upregulation [35, 37, 41]. However, these studies relied predominantly on calcium imaging, which captures slower suprathreshold events and different components than electrophysiology, or c‐Fos expression, which is limited to correlated activity across large temporal timescales. In contrast, our experiments show that MENPs can be activated using pure static magnetic stimulation, without any AC component, to modulate spontaneous activity in an intact recurrent cortical network. Achieving robust modulation with <100 mT SMS is unprecedented, as prior MENP activation typically required AC waveforms above coercivity [42]. The ability of MENPs to reshape Up/Down state dynamics under static fields suggests either enhanced sensitivity of intact microcircuits or cooperative effects arising from the recurrent connectivity of the cortical network.

Since one of the main concerns about MENPs is their biocompatibility, we also assessed their effect on SO activity while inactive. We observed that, before magnetic activation, the administration of inactive MENPs suspended in PBS did not disrupt the fundamental slow‐oscillatory architecture, as slices continued to express the hallmark alternation between Up and Down states. The global spatiotemporal organization of the slow oscillation remained intact, indicating that inactive MENPs do not disrupt the core rhythm‐generating mechanisms of the network. This is consistent with the established biosafety profile of CFO‐BTO MENPs, which exhibit low cytotoxicity and minimal inflammatory response across multiple in vitro and in vivo studies [35, 37, 42]. For this reason, in all subsequent experiments probing the effects of activating MENPs with SMS, we used the stable slow‐oscillation pattern recorded after the 1‐h incubation with MENPs as the functional baseline. Overall, these observations support that the neuromodulatory effects reported in this work arise specifically from magnetoelectric activation under SMS, rather than from passive exposure to MENPs or their carrier solution.

This study has not determined the subcellular localization of MENPs within the cortical slices. However, MENPs were applied to the slice surface in an interface chamber and allowed to stabilize for 1 h, suggesting predominant distribution over superficial tissue layers with possible passive penetration. The persistence of the physiological effects under continuous ACSF perfusion indicates that the particles remained sufficiently associated with the tissue to modulate network activity. At present, localized stimulation at the neuronal membrane should therefore be considered a biophysical inference based on the nanoscale dimensions of MENPs and computational modeling, and not an experimentally mapped mechanism. Future studies combining high‐resolution imaging (e.g., TEM), functional recordings and modeling will be necessary to determine MENP localization relative to neural elements and to fully understand the underlying mechanisms at the cellular level.

Together, our results highlight the potential of MENPs to support wireless, spatially targeted neuromodulation, provided that particle delivery or activation can be spatially restricted. By amplifying weak magnetic fields into bioelectrically active signals, MENPs offer a promising platform for influencing cortical dynamics in vitro and, potentially, in vivo. Such targeted modulation of cortical excitability could be particularly beneficial in disorders characterized by disrupted oscillatory dynamics, such as stroke [47] or epilepsy [51]. This proof‐of‐concept study provides new insight into MENP‐based modulation of network rhythms and sets the stage for future investigations into clinical and translational applications of magnetoelectric nanotechnology in neuroscience. It lays the groundwork for exploring MENP‐based neuromodulation not only as a research tool, but also as a minimally invasive therapeutic strategy.

## 4 Conclusion

MENPs convert weak (<100 mT) and neutral static magnetic fields into localized electric perturbations that act on neurons, and their effects reverberate through the network, resulting in a macroscopic modulation of emergent cortical dynamics. Consistent with our experimental observations, magnetoelectric modeling predicts that MENPs exposed to the same magnetic field strengths generate non‐negligible local electric fields. Our results demonstrate that local electric fields generated by MENPs can increase the local network excitability through CFO‐BTO MENP‐specific magnetoelectric mechanisms. The present experiments support the possibility that spatially restricted MENP delivery or activation could enable targeted neuromodulation, which should be further tested directly in future studies. By allowing weak, localized fields to modulate neuronal activity, in ways inaccessible to conventional approaches, stimulation using MENPs opens new avenues for precise, non‐invasive neuromodulation and potential therapeutic strategies for neurological disorders.

## 5 Experimental Methods

### 5.1 Ethics Statement

All experimental procedures were conducted in accordance with institutional and European guidelines for the care and use of laboratory animals. Protocols were approved by the Animal Experimentation Ethics Committee of the University of Barcelona and complied with Spanish (RD53/2013) and European Union (2010/63/EU) regulations.

### 5.2 Ferret Cortical Slice Preparation

Coronal cortical slices were prepared following established procedures for ferret visual cortex slices, adapted from Sánchez‐Vives and McCormick [53] and incorporating the sucrose‐substitution technique of Aghajanian and Rasmussen [79]. Adult ferrets (4–7 months old, either sex; *n* = 27; Euroferret, Denmark) were deeply anesthetized with an intramuscular injection of ketamine (8 mg kg^−1^) and medetomidine (0.1 mg kg^−1^). Depth of anesthesia was confirmed by the absence of limb‐withdrawal reflexes before decapitation. The forebrain was rapidly removed and immersed in ice‐cold (4°C–10°C), carbogenated sucrose‐based cutting solution (in mM: sucrose 213, KCl 2.5, NaH_2_PO_4_ 1, NaHCO_3_ 26, CaCl_2_1, MgSO_4_ 3, glucose 10). Coronal slices (400 µm) containing primary and secondary visual cortical areas (areas 17, 18, and 19) were obtained using a vibrating microtome (7000smz‐2, Campden Instruments, UK) (Figure 2A). To enhance tissue viability, slices were initially incubated for 30 min in an interface‐style chamber (Scientific Systems Design Inc., Canada) containing a 1:1 mixture of sucrose‐substituted solution and ACSF. Slices were then perfused with ACSF for 1 h, followed by at least 40 min in an in vivo–like modified ACSF before recordings were taken. Standard ACSF contained (in mM): NaCl 126, KCl 2.5, MgSO_4_ 2, NaH_2_PO_4_ 1, CaCl_2_ 2, NaHCO_3_ 26, glucose 10. The in vivo–like ACSF had identical composition except for (in mM): KCl 4, MgSO_4_1, and CaCl_2_ 1. All solutions were continuously bubbled with 95% O_2_/5% CO_2_ to maintain pH 7.4. Slices were kept at 34.5°C–36.0°C throughout the experiment. Electrophysiological recordings began only after completion of the recovery period in the in vivo–like ACSF.

### 5.3 Electrophysiological Recordings

LFPs were recorded using custom‐made flexible black‐platinum microelectrode arrays (MEAs) (16‐channel; CNM, Barcelona, Spain). Signals were amplified in two stages: first ×10 using an MPA8I amplifier, and subsequently ×100 using a PGA16 amplifier (Multi Channel Systems, Reutlingen, Germany). The amplified signals were digitized at 10 kHz through a Power1401 interface (CED, Cambridge, UK) and acquired using Spike2 software (CED, Cambridge, UK). Quantitative analyses were restricted to channels exhibiting stable spontaneous activity and reliable Up‐state detection with adequate signal‐to‐noise ratio.

### 5.4 Synthesis and Characterization of MENPs

MENPs were synthesized following a wet‐chemical to sol‐gel pathway using established protocols [42, 43]. Particles were coated with polyethylene glycol to reduce agglomeration and dispersed in PBS prior to transport.

### 5.5 Computational Model

To evaluate the magnetoelectric properties of the core‐shell nanoparticles involved in the experimental studies, we applied a previously built and validated 2D axisymmetric computational model developed in COMSOL Multiphysics [34, 80], which couples magnetic fields, solid mechanics, and electrostatics modules through the magnetostriction and piezoelectric Effect multiphysics couplings. The model, which accounts for nonlinear magnetic behavior via the Jiles‐Atherton hysteresis formulation, was adapted to the specific geometry of the nanoparticles synthesized for these in vitro experiments: cobalt ferrite (CFO) cores with a diameter of 15 nm, coated with a 2 nm barium titanate (BTO) shell.

### 5.6 Magnetic Field Calibration

The static magnetic field generated by the neodymium magnets was characterized prior to slice experiments. Four cylindrical neodymium (N42) rod magnets (1 cm length × 1 cm diameter, Distrelec Schweiz AG) were stacked to produce the applied field. Field measurements were performed using an MF‐30K AC/DC Gauss meter (Latnex, Canada; range 0–3000 mT/0–30 000 G). Calibration involved quantifying the magnetic flux density at incremental distances perpendicular to the magnet surface for both polarities (north and south). For experiments, a minimum distance of 0.5 cm from the slice surface was selected, ensuring safe operation without obstructing oxygen flow. At this distance, the magnetic flux density reached 93.4 mT (Figure 2E), which corresponds to the effective field experienced by the cortical slices during stimulation.

### 5.7 Static Magnetic Stimulation Without MENPs

To assess whether the applied static magnetic field alone modulates cortical activity, four stacked neodymium magnets were slowly positioned perpendicular to the slice using a micromanipulator (Narishige, Japan) to a final distance of 0.5 cm from the slice surface. The magnets were held in place for up to 100 s. LFPs were recorded continuously throughout the stimulation protocol, including the period before, during, and following stimulation (Figure 2C). The procedure was then repeated with reversed polarity (north/south), as defined by the MF‐30K AC/DC Gauss meter. These trials served as controls to evaluate the effect of the DC magnetic field in the absence of MENPs.

### 5.8 MENP Application

MENPs were suspended in PBS at 1 µg µL^−1^. Two 6 µL drops of this suspension (total 12 µg) were carefully applied to the surface of the slice using a micropipette. Because the electrode array covered part of the slice surface (Figure 3A,B), MENPs reached only the tissue not covered by the array. Slices were incubated for 1 h to allow particle integration. LFP activity was recorded after MENP application to verify that inactive MENPs alone did not alter ongoing cortical activity.

### 5.9 MENP + Static Magnetic Stimulation

Following MENP incubation, the same DC magnetic stimulation protocol described above (0.5 cm distance, ∼100 s per polarity) was applied. LFP activity was continuously recorded before, during, and after stimulation to assess polarity‐dependent modulation and post‐stimulation dynamics in the presence of MENPs (Figure 2D). Hereafter, the activity recorded before stimulation is referred to as the baseline condition.

### 5.10 Up State Detection

Up and Down states were detected as described by Dasilva et al. [46]. Three signals were extracted from the raw LFP: (i) slow oscillation deflection (SO), (ii) gamma‐band envelope (variance of the 30–80 Hz signal [81]), and (iii) local network firing (MUA; power in 200–1500 Hz band, with 5 ms windows [82, 83, 84, 85]). MUA was logarithmically scaled (logMUA) to reduce high fluctuations. The three z‐scored signals were combined using principal component analysis (PCA), and the first component, which showed a bimodal distribution, was used to define thresholds separating Up and Down states.

### 5.11 Metrics Derived from Up State Detection

For each slice, a minimum of two recording sites were analyzed because cortical slow oscillations constitute highly synchronized network events. Representative channels with optimal signal quality were considered sufficient to characterize the overall network dynamics. Although activity was monitored across the full MEA array, no dedicated spatial propagation analysis was performed in the present study. Event durations (Up and Down states) and SO frequency were quantified. SO frequency (Up events/s) was quantified as the total number of detected Up states divided by the total recording duration for each trial. For each condition, the mean Up state duration, Down state duration, and SO frequency were computed across all events and channels. Outliers in event durations and SO frequency, attributed to recordings with a very low number of Up states, were excluded by calculating the 95% confidence interval, respectively, for each condition and polarity, with values outside this range discarded. Event durations were paired across experimental stages by matching recordings with identical slice ID, stimulus ID, and event type. Pairing was performed independently for north and south magnetoelectric polarities, aligning each pre‐stimulation condition (baseline) with the corresponding stimulation (SMS N/S) and post‐stimulation (Post SMS N/S) conditions. Changes in event duration (Δduration, in seconds) were calculated as the difference between the stimulation or post‐stimulation duration and the corresponding baseline duration.

### 5.12 Firing Rate Computation

Firing rates were derived from the absolute value of the logMUA signal. For each Up and Down state, the mean firing rate was calculated as the average logMUA within the corresponding epoch. To characterize the temporal structure of firing around state transitions, instantaneous firing rates were computed by aligning logMUA to each Up or Down onset. A temporal window around each transition was extracted and smoothed with a Gaussian kernel.

### 5.13 Statistical Analysis

Statistical analyses were performed in Python using SciPy and statsmodels. Because several outcome variables exhibited non‐Gaussian distributions and the number of biological replicates (cortical slices) was limited, non‐parametric statistical approaches were used for analyses performed at the slice level. Unless otherwise stated, data are presented as mean ± standard deviation (SD). Statistical significance thresholds were defined as *p* < 0.05 (^*^), *p* < 0.01 (^**^), *p* < 0.001 (^***^), and *p* < 0.0001 (^****^).

For comparisons between independent experimental groups at the slice level, two‐sided Mann–Whitney U tests were used. For repeated‐measures comparisons involving the same slices across two experimental conditions, two‐sided Wilcoxon signed‐rank tests were applied. When more than two repeated conditions were compared within the same slices, Friedman tests were first used as omnibus repeated‐measures tests, followed by pairwise Wilcoxon signed‐rank post hoc comparisons. Multiple‐comparison correction was performed using the Benjamini–Hochberg false discovery rate (BH‐FDR) procedure.

For analyses involving repeated trial‐level measurements obtained from the same cortical slice, linear mixed‐effects models (LMMs) were used to account for the hierarchical structure of the data and avoid pseudoreplication. In these models, experimental condition was treated as a fixed effect and slice identity (defined by slice label and experimental date) was included as a random intercept to account for correlations among trials originating from the same slice. Models were fitted using restricted maximum likelihood estimation (REML). Only slices present in all compared experimental conditions were included in each repeated‐measures analysis to preserve within‐slice pairing.

This mixed‐effects framework was applied to analyses of slow‐oscillation frequencies and state‐duration metrics in Figure 5, where multiple trials were recorded from each slice under different stimulation conditions. Compared with analyses based solely on slice‐averaged values, LMMs preserve trial‐level variability while appropriately modeling the non‐independence of repeated measurements obtained from the same biological preparation.

In one comparison for which the mixed‐effects model failed to converge because of singular covariance estimation, statistical inference was instead performed using a paired Wilcoxon signed‐rank test on slice‐averaged values.

All reported p‐values were two‐sided. Unless otherwise indicated, multiple‐comparison correction throughout the study was performed using the Benjamini–Hochberg false discovery rate procedure.

## Author Contributions

**Nathalia Cancino‐Fuentes**: conceptualization, methodology, data curation, investigation, validation, formal analysis, visualization, writing – original draft, writing – review and editing. **Joana Covelo**: writing – original draft, writing – review and editing, conceptualization, methodology, investigation, visualization. **Anton Guimera‐Brunet**: methodology. **Alejandro Suarez‐Pperez**: conceptualization, methodology, data curation, investigation, validation, formal analysis, visualization, writing – original draft, writing – review and editing. **Xavi Illa**: conceptualization, methodology. **Vitaly Pustovalov**: methodology. **Elric Zhang**: conceptualization, methodology, investigation, validation, writing – review and editing. **Marta Parazzini**: conceptualization, methodology, software, supervision, funding acquisition, resources, writing – review and editing. **Marta Bonato**: conceptualization, methodology, software, investigation, validation, writing – review and editing. **Hao Ye**: methodology, investigation. **Maria V. Sanchez‐Vives**: supervision, funding acquisition, conceptualization, methodology, project administration, resources, writing – review and editing. **Valentin Gantenbein**: methodology. **Salvador Pané**: conceptualization, methodology, supervision, funding acquisition, writing – review and editing. **Cagatay M. Oral**: methodology.

## Funding

This work was funded by META‐BRAIN, a project with Grant Agreement No 101130650 funded by the European Innovation Council and SMES Executive Agency (EISMEA – European Union) to MVSV, MP, XI, SP. Funded by Project INFRASLOW PID2023‐1529180B‐100 and by ERC, NEMESIS, 101071900 to MVSV. Funded by Swiss Secretariat for Education Research and Innovation (SERI) under the frame of the EU Horizon Europe Research and Innovation Programme (EVA project GA No. 101047081) to SP.

## Conflicts of Interest

The authors declare no conflicts of interest.

## Supporting information




**Supporting File**: advs76867‐sup‐0001‐SuppMat.pdf.

## Data Availability

Data is available from corresponding author upon reasonable request.
