# Supplementary material for: Magnetoelectric Nanoparticles Enable Modulation of Cortical Networks by Low‐Intensity Static Magnetic Fields In Vitro
Source: Adv Sci (Weinh). 2026 Jul 31:e76867. Online ahead of print. doi: 10.1002/advs.76867 (PMC13426097; doi:10.1002/advs.76867)
Supplement: Supplementary file 1 — Supporting File: advs76867‐sup‐0001‐SuppMat.pdf. [file ADVS-9999-e76867-s001.pdf]

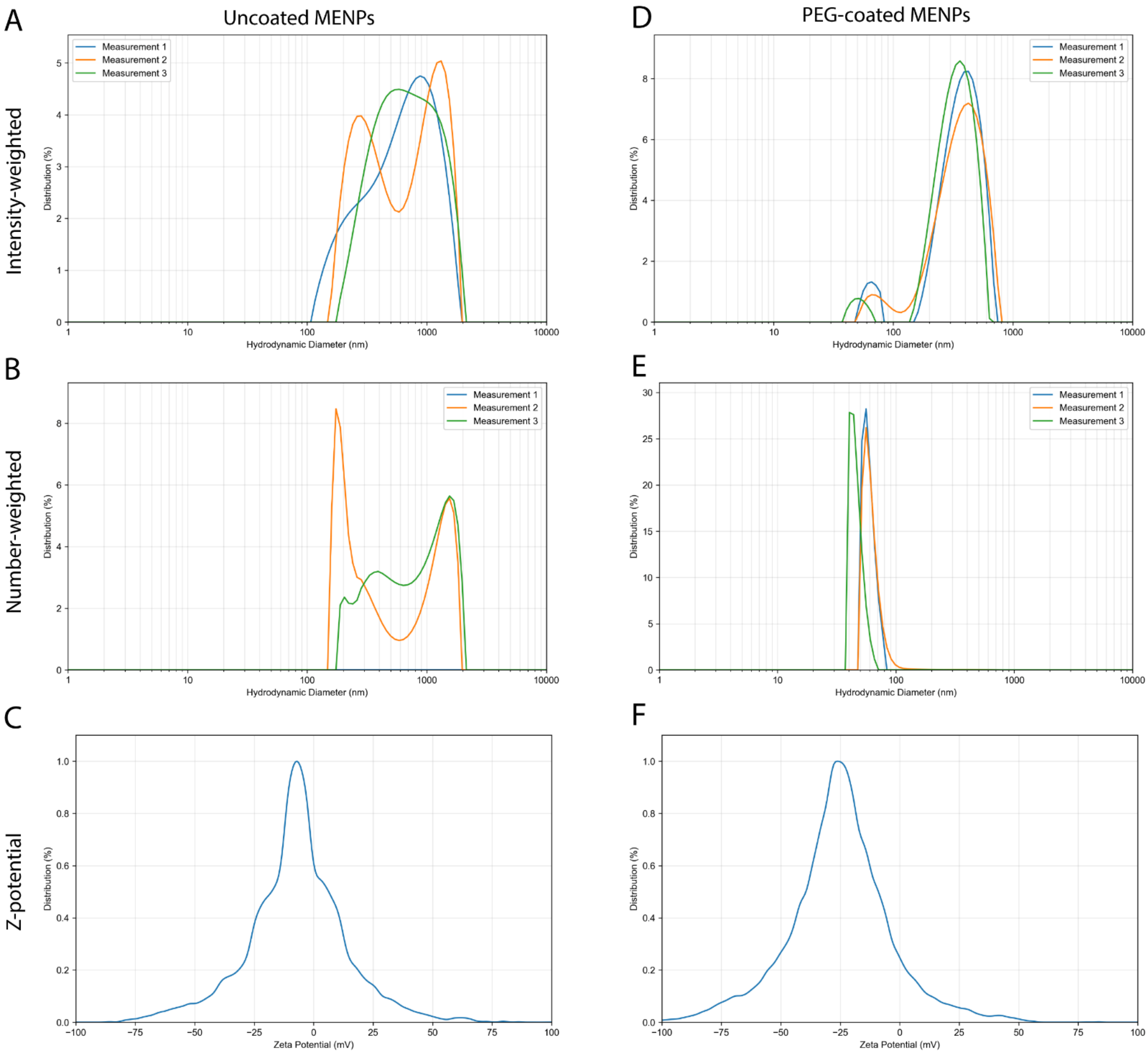

Supplementary Figure 1 | Colloidal characterization of uncoated and PEG-coated magnetoelectric nanoparticles. (A–F) Dynamic light scattering (DLS) and zeta potential measurements of magnetoelectric nanoparticles (MENPs) dispersed in phosphate-buffered saline (PBS), corresponding to the same batch used in electrophysiological experiments. Panels (A–C) correspond to uncoated MENPs and panels (D–F) to PEG-coated MENPs.(A, D) Intensity-weighted hydrodynamic diameter distributions (x-axis: hydrodynamic diameter, nm; y-axis: distribution, %). (B, E) Number-weighted hydrodynamic diameter distributions (x-axis: hydrodynamic diameter, nm; y-axis: distribution, %). (C, F) Zeta potential distributions (x-axis: zeta potential, mV; y-axis: distribution, %). For DLS measurements (A, B, D, E), three independent measurements are shown for each condition, overlaid within each panel. All measurements were performed in PBS under identical conditions to those used for slice application.
